# Supplementary material for: Novel Biomarkers for Inflammatory Bowel Disease and Colorectal Cancer: An Interplay between Metabolic Dysregulation and Excessive Inflammation
Source: Int J Mol Sci. 2023 Mar 22;24(6):5967. doi: 10.3390/ijms24065967 (PMC10055751; doi:10.3390/ijms24065967)
Supplement: Supplementary file 1 [file ijms-24-05967-s001.zip › ijms-2155570 Supplementary Figures Final (Dr Baksh update).pdf]

Novel Biomarkers for Inflammatory Bowel Disease and Colorectal Cancer: An interplay Between Metabolic Dysregulation and Excessive Inflammation

Mohamed Salla[1,9*], Jimmy Guo[2*], Harshad Joshi[2] Marilyn Gordon[3], Hitesh Dooky[1], Justine Lai[4], Samantha Capicio[1], Heather Armstrong[3,#], Rosica Valcheva[2], Jason R. B. Dyck[3,5,6], Aducio Thiesen[7], Eytan Wine[3,8], Levinus A. Dieleman[2] and Shairaz Baksh[1,3,4,10,11]

Biochemistry[1], Departments of Medicine[2], Pediatrics[3], Oncology[4], Pharmacology[5], Cardiovascular Research Centre[6], Lab Medicine and Pathology[7], Physiology[8], Faculty of Medicine & Dentistry, University of Alberta, 113 Street 87 Avenue, Edmonton, AB, Canada, T6G 2E1

[9]Current affiliation and address: Lebanese International University, Department of Biological & Chemical Sciences, Bekaa Campus, Khiyara – West Bekaa, Lebanon

[10]Member, Cancer Research Institute of Northern Alberta and Women and Children's Health Research Institute, 113 Street 87 Avenue, University of Alberta, Edmonton, AB, Canada, T6G 2E1.

[11]BioImmuno Designs, Inc., 4747 154 Avenue NW, Edmonton, AB. Canada. T5Y 0C2

[#]Current affiliation and address: Assistant Professor and Tier 2 CRC; Department of Internal Medicine, Manitoba Centre for Proteomics and Systems Biology; University of Manitoba

Room 808 John Buhler Research Centre; 715 McDermot Avenue, Winnipeg, MB, R3E 3P4

[*]Equal authorship to this study

Correspondence and co-senior authors: Dr. Shairaz Baksh, E-mail: sbakshBID@gmail.com, Tel: 0017802390518 and Dr. Levinus A. Dieleman, l.dieleman@ualberta.ca, Tel: 0017804921888

Supplementary Figures

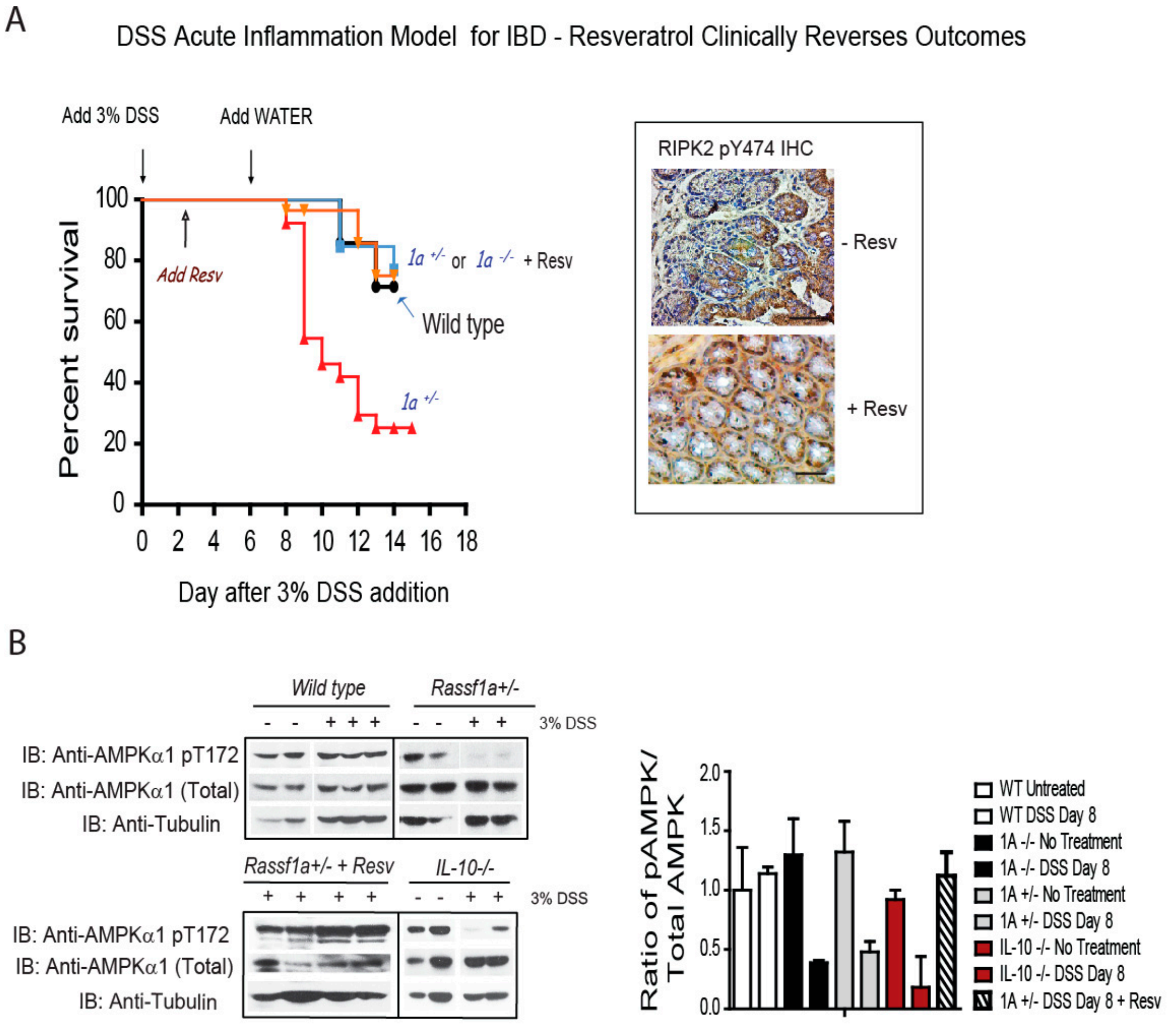

Supplementary Figure S1. Resveratrol-directed therapeutic intervention of IBD in a mouse model. DSS-induced inflammation injury model of acute IBD was carried out in the DSS-susceptible model, *Rassf1a*$^{+/-}$ (*1a*$^{+/-}$). Animals were treated with 3% DSS in the drinking water for 7 days followed by 7 days of recovery. Resveratrol was given (as indicated) as food pellets at 1 g/kg resveratrol in the diet in (A). These mice consumed ~ 3 g/ day = human equivalent dose [HED] of 1 mg resveratrol/day for a 70 kg person or 2 bottles of red wine/day). At day 8, animals were euthanized and colon lysates prepared and analyzed for the indicted proteins and tissue section. Mice were monitored for piloerection, bloatednesss, tremors, lack of movement, and rectal bleeding. (A) Left panel, Kaplan-Meier curve with N = 15-20 animals were used for all conditions and p-value between wild type and *Rassf1a*$^{-/-}$. was < 0.001. Right panel, representative descending colon sections from experiment in the left panel immunostained with RIPK2 pY 474. (B) Left

panel, immunoblot of descending colon lysates from experiment in A with the indicated antibodies. In addition, results for AMPK staining was carried out for DSS treatment in *Il-10*$^{-/-}$ mice as indicated. This result was quantified in the right panel. P value < 0.003 for all comparative categories with n = 2-4.

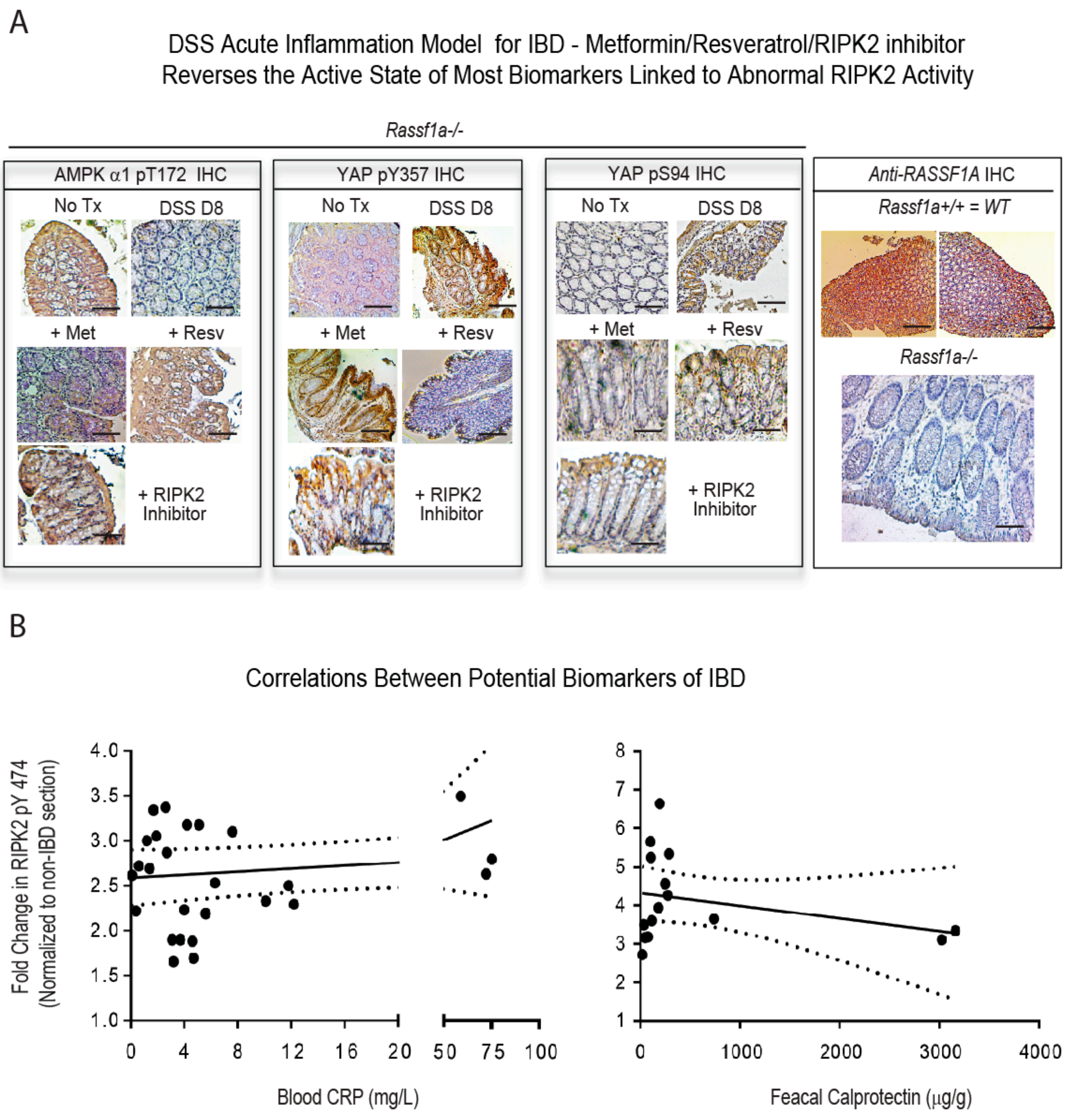

Supplementary Figure S2. (A) Immunohistochemical staining of identified biomarkers upon metformin, resveratrol or RIPK2 inhibitor treatment in a DSS-inflammation mouse model. For each panel, representative sections are shown utilizing the *Rassf1a*$^{-/-}$ colonic tissue sections from -/+ DSS treated animals as outlined in the experimental protocols. All images taken at 10X magnification. (B) Correlations between biomarkers. For left graph, $r^2 = 0.065$, p value = 0.191 and n = 31; right panel, $r^2 = 0.09$, p value = 0.278 and n = 16.

A

Detection of Active RIPK2 (pY 474 IHC)

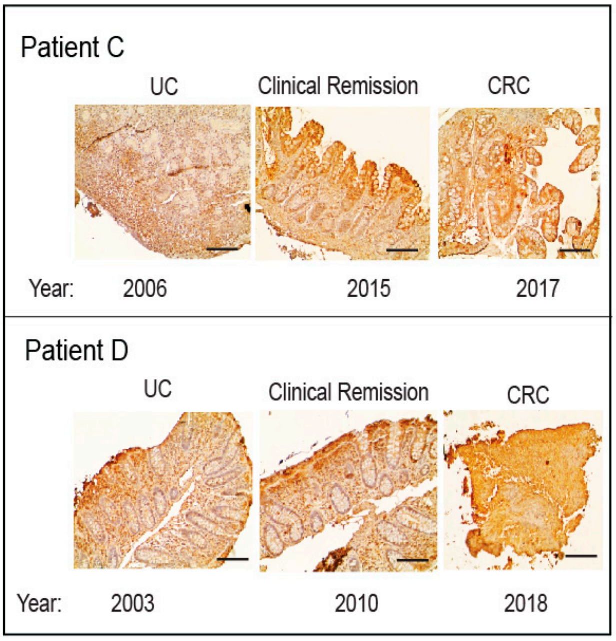

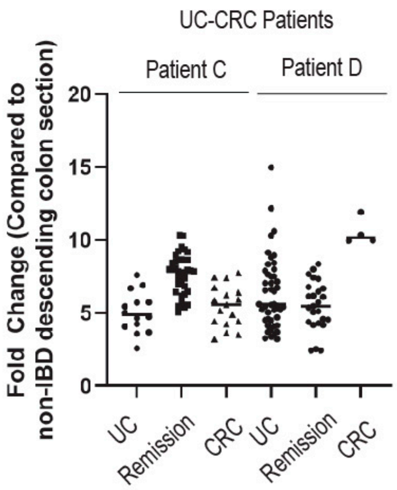

B

Detection of Active YAP (pS94 IHC)

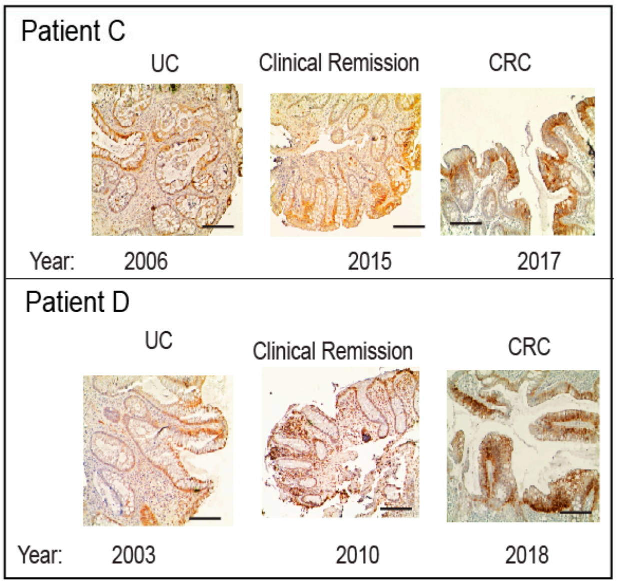

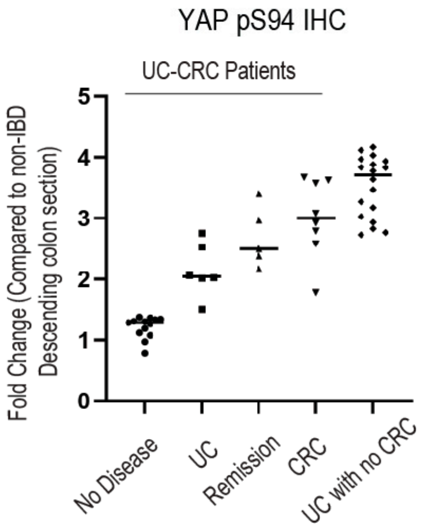

C

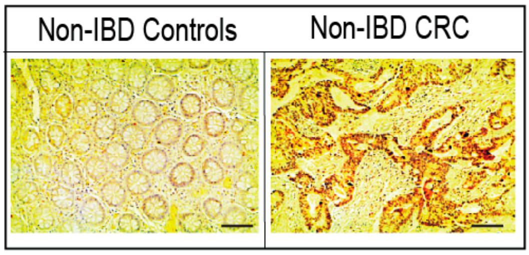

Detection of Active YAP (pS94 IHC)

Supplementary Figure S3. Immunohistochemical staining for RIPK2 (A) and YAP pS94 (B) in two more UC-CRC case study patients (A, B) and controls/non-IBD CRC sections (C). Included in this panel is stage of disease and year diagnosed. Patient D have since passed away of CRC related disease. Left panels, representative sections for each category are shown; Right panels, summary of fold changes for both RIPK2 pY 474 and YAP pS94. For A (Patient C), P value < 0.0001 between UC and Remission, P value < 0.95 for difference between UC and CRC and P value < 0.0001 between Remission and CRC (n = 4 - 36 sections). For A (Patient D), P value < 0.12 between UC and Remission, P value < 0.02 for difference between UC and CRC and P value < 0.0001 between Remission and CRC (n = 4 - 36 sections). For B, P value < 0.0001 between UC and UC with no CRC, P value < 0.0894 for difference between UC and UC in remission and P value < 0.02 between UC and CRC (n = 6-9 sections). For (C), representative sections are shown for YAP pS94 on non-IBD controls and for CRC.
